# Hypothalamic tanycytes—masters and servants of metabolic, neuroendocrine, and neurogenic functions

**DOI:** 10.3389/fnins.2015.00387

**Published:** 2015-10-29

**Authors:** Timothy Goodman, Mohammad K. Hajihosseini

**Affiliations:** School of Biological Sciences, University of East AngliaNorwich, UK

**Keywords:** tanycytes, postnatal neurogenesis, barrier function, neuroendocrine, appetite and energy expenditure

## Abstract

There is a resurgent interest in tanycytes, a radial glial-like cell population occupying the floor and ventro-lateral walls of the third ventricle (3V). Tanycytes reside in close proximity to hypothalamic neuronal nuclei that regulate appetite and energy expenditure, with a subset sending projections into these nuclei. Moreover, tanycytes are exposed to 3V cerebrospinal fluid and have privileged access to plasma metabolites and hormones, through fenestrated capillaries. Indeed, some tanycytes act as conduits for trafficking of these molecules into the brain parenchyma. Tanycytes can also act as neural stem/progenitor cells, supplying the postnatal and adult hypothalamus with new neurons. Collectively, these findings suggest that tanycytes regulate and integrate important trophic and metabolic processes and possibly endow functional malleability to neuronal circuits of the hypothalamus. Hence, manipulation of tanycyte biology could provide a valuable tool for modulating hypothalamic functions such as energy uptake and expenditure in order to tackle prevalent eating disorders such as obesity and anorexia.

## Introduction

The role and importance of tanycytes in diverse physiological functions—from barrier properties to neuroendocrine, metabolic, and neurogenic processes has been the subject of several excellent reviews (Rodríguez et al., 2005; Bolborea and Dale, 2013; Cheng, 2013; Coll and Yeo, 2013; Langlet, 2014; Lee and Blackshaw, 2014; Sousa-Ferreira et al., 2014; Ebling, 2015). In this brief review we aim to provide an update, integrate our knowledge of tanycyte ontogeny with physiology, and critically discuss the intricacies and challenges in understanding their biology.

## Origin, organization and tanycyte subtypes in the hypothalamus

Tanycytes of the adult brain are regarded as residual radial glial cells. Radial glial cells, and subtypes therein (De Juan Romero and Borrell, 2015), are prominent in the developing central nervous system (CNS) and serve at least five important functions: they act as neural stem/progenitors cells; provide a scaffold for migration of newly formed neurons to their final position; play an important role in gyrification of the cerebral cortex; demarcate the boundary between some axonal tracts and the adjacent white matter; and, at the end of embryonic neurogenesis, a subset differentiate into astrocytes. After birth, Radial glial cells persist in discrete regions of the CNS, namely, as Muller glia of the retina, Bergmann glia of the cerebellum and in some circumventricular organs (CVO: the Subfornical and subcommisural organs; Pineal gland; Organum vasculosum of the lamina terminalis; Area postrema; and the Median Eminence; Bennett et al., 2009; Morita et al., 2015). Likely, some of the embryonic Radial glial cells' functions are conserved in adult tanycytes.

The term tanycytes (“cells with drawn out process”) was first coined by Horstmann in 1954 and although some early literature used it to describe Radial glial-like cells found in CVOs, in recent times, it has become synonymous mostly with hypothalamic Radial glia.

Hypothalamic tanycytes emerge during late embryonic development, in rats around embryonic day 19 (equivalent to E17 in the mice), from similar if not the same neurogenic ventricular zone cells that generated hypothalamic neurons a few days earlier (Altman and Bayer, 1978). Tanycytes occupy the floor and ventro-lateral walls of the third ventricle (3V) and are distinguished from more dorsally-located cuboidal ependymal cells by their long radial processes, as well as lack of beating cilia. Nonetheless, a transition zone in which ependymal cells interdigitate with tanycytes is observed in the mature 3V ependymal layer (Hajihosseini et al., 2008). Recent findings show that specification of hypothalamic tanycytes is regulated in part by transcription factor Lhx2 acting upstream of the gene Rax, since, in the absence of Lhx2, presumptive tanycytes exhibit ependymal cell features (Salvatierra et al., 2014).

**Figure 1:**
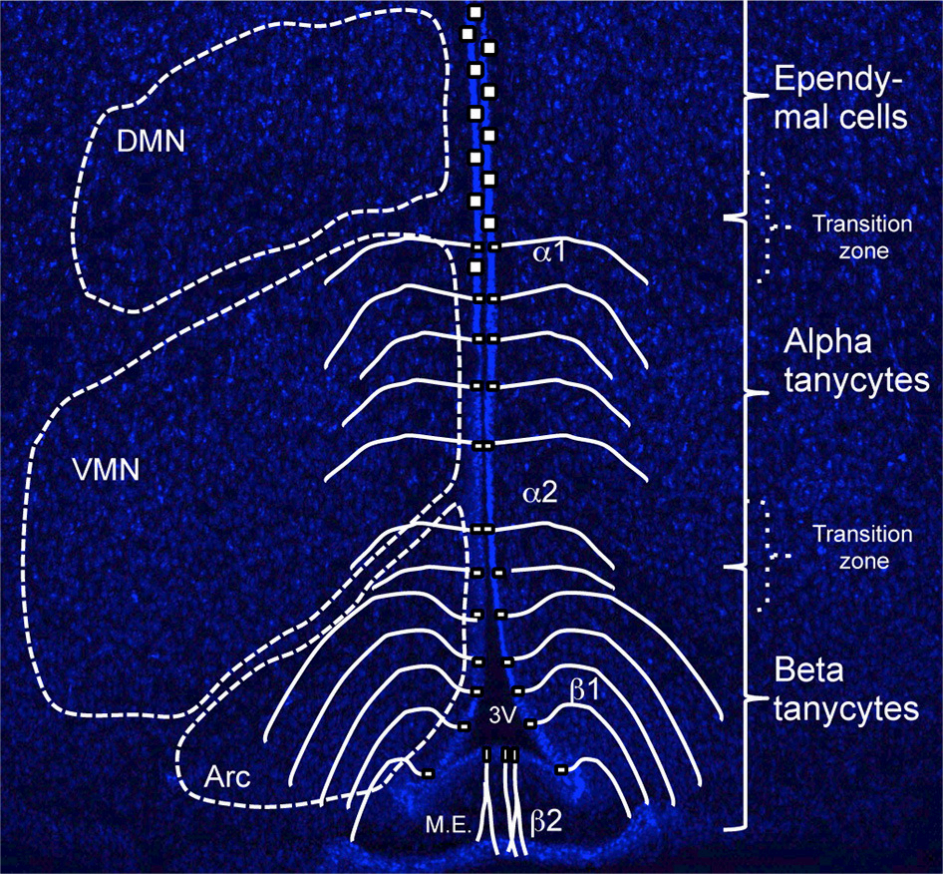
**Organization and distribution of hypothalamic tanycytes**. Coronal section of adult hypothalamus at approximate bregma -1.5 labeled with Hoechst to highlight parenchymal neuronal nuclei, Dorsomedial (DMN), Ventromedial (VMN), and Arcuate (Arc) that flank the third ventricle (3V) at this bregma. Superimposed is a schematic depicting the domains of tanycyte subtype (β vs. α) and ependymal cells. Note that the dorso-ventral extent of these domains, as well as the flanking parenchymal nuclei varies depending on bregma level (see Figure 2). M.E., Median Eminence.

Tanycytes have been further subdivided into alpha (α) and beta (β) subtypes and although both have a radial appearance, several characteristics distinguishes them apart (Rodríguez et al., 2005; Figure 1). First, β-tanycytes (further subdivided in to β1 and β2) occupy the floor and more ventral parts of the 3V ependyma, whilst α-tanycytes (α2 and α1) reside more dorsally to β-tanycytes (Figure 1). However, as with the ependymo-tanycyte transition, similar transition zones are observed between different tanycyte subtypes, and this makes it particularly difficult to reliably isolate each subtype through microdissection of fresh brain preparations. Second, β-tanycyte cell processes arch back medially and ventrally to contact the pial surface, the portal blood vessels of the median eminence (β2) or the hypothalamic parenchymal capillaries, and, to segregate the arcuate nucleus from the median eminence and its milieu (β1). By contrast, most α-tanycyte processes lack blood vessel contact, terminating in close proximity of parenchymal neurons (Rodríguez et al., 2005). Thus, visualizing the entire process projection of tanycytes can aid in their sub-classification *in vivo*. It has also been reported that most β, but only a few α-tanycytes, carry primary cilia (Miranda-Angulo et al., 2014), presumably enabling β-tanycytes to a respond to cilia-mediated signaling molecules/pathways such as sonic hedgehog (Han et al., 2008). Conversely, whereas α-tanycytes project a single villus, β-tanycytes project multi-villi into the 3V space. Interestingly, β2-tanycytes are the only subtype to express N-Cadherin and Calveloin-1 (Peruzzo et al., 2004). The latter has been implicated in endocytosis and recycling of growth factor receptors as a potential mechanism to regulate levels of cell signaling, and this concurs with β-tanycyte restricted expression of several growth factor genes, such as FGF-receptors 1, 2 and CNTF-receptor (Table 1). Lastly, processes of β- but not α-tanycytes receive direct neural input, particularly from GnRH neurons (Rodríguez et al., 2005). These differences suggest that β- and α-tanycytes have fundamentally different biological properties (see also below).

**Table 1:**
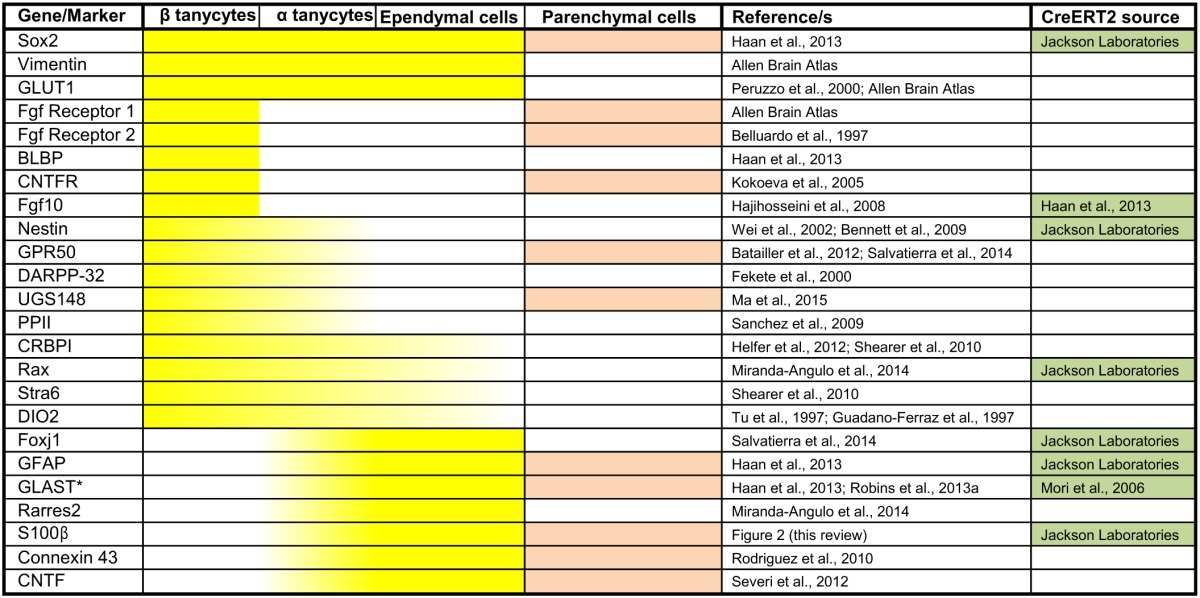
**Common and domain-restricted gene expression within the cell lining of the adult third ventricle**.

A broad survey of published images (in situ hybridization; immunolabeling; CreERT2-mediated reporter activation) shows that only a handful of genes are expressed throughout the ependymal lining, with most other genes being either restricted to β- or α-tanycytes and ependymal cells. However, gradients of expression are also evident as indicated by fading colors, possibly reflecting the transition zones between the various cell domains. As indicated, some genes show additional expression in the flanking parenchymal cells. The availability and source of the relevant CreERT2 mice is also indicated in the last column. ^*^For GLAST, acute CreERT2 reporter pattern is indicated, which contrasts with reported immunoreactivity in β as well as α tanycytes by Berger and Hediger (2001). Sox2, Sex determining region Y-box 2; GLUT1, glucose transporter 1; BLBP, brain lipid-binding protein; CNTFR, ciliary neurotrophic factor receptor; GPR50, G protein-coupled receptor 50; DARPP-32, dopamine and cAMP-regulated neuronal phosphoprotein of 32 kDa; PPII, pyroglutamyl peptidase II; CRBPI, cellular retinol binding protein I; Rax, retina and anterior neural fold homeobox transcription factor; Stra6, stimulated by retinoic acid 6; DIO2, type 2 iodothyronine deiodinase; Foxj1, forkhead box protein J1; GFAP, glial fibrillary acidic protein; GLAST, glutamate aspartate transporter; Rarres2, retinoic acid receptor responder (tazarotene induced) 2.

From a developmental view, it is not clear exactly when or how does the β- vs. α-tanycyte diversification occur and/or whether there is a lineage hierarchy, or a to-and-fro flux between the two subtypes. The latter would depend on the level of fluidity within the 3V ependymal layer, which has been observed in other CNS VZ compartments (Walsh and Cepko, 1993). However, α2-tanycytes are developmentally more advanced than α1 in rodents, and embryonically born tanycytes acquire their adult characteristics during the first postnatal month (Rodríguez et al., 2005).

**Figure 2:**
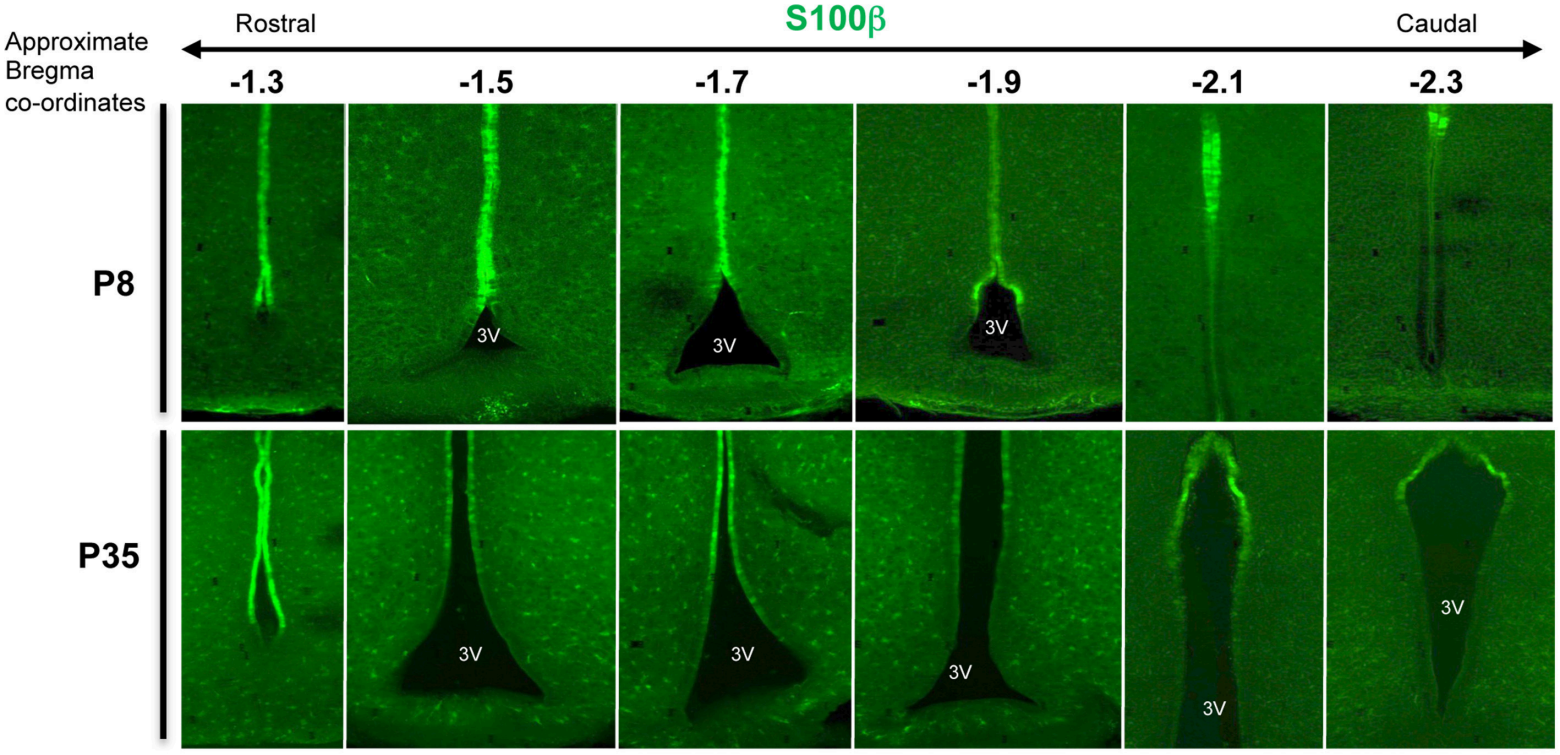
**Varying dorso-ventral S100β expression in the 3V ependymal lining**. Coronal section of mouse hypothalamus at postnatal days 8 (P8) and P35, immunolabeled with anti-calcium binding protein, S100β antibodies, which largely encompasses the domain of α-tanycytes and ependymal cells. Note the varying dorso-ventral extent of this marker at different bregma levels (here from –1.3 to –2.3), and the additional punctate expression by glial cells/glial progenitors within the neighboring parenchyma.

A broad survey of published literature and gene expression atlases (e.g., Allen Brain atlas, www.brain-map.org) shows that whilst adult β- and α-tanycytes share some markers (e.g., expression of Sox2 and Vimentin), there is also a class of β-tanycyte—specific genes/markers. Indeed, generally, the gene/marker profile of α-tanycytes is more resemblant of more dorsal non-tanycytic ependymal cells than β-tanycytes (Table 1). For example, throughout postnatal life, the glial cell marker S-100β which is largely absent from the β-tanycyte domain, is expressed by α-tanycytes and all other 3V ependymal cells. Interestingly, whilst the walls of the adult 3V and its flanking hypothalamic parenchyma span bregma co-ordinates 0.50 to –2.6 mm along the rostro-caudal axis, the distribution and abundance of tanycytes is much more restricted along this axis (Haan et al., 2013), but also is more dynamic dorso-ventrally. The latter is exemplified by the varying dorso-ventral extent of S-100β within the ependymal layer at different bregma co-ordinates (Figure 2).

The selective and differential marker gene expressions highlighted in Table 1 likely endow different properties to ependymal cells vs. tanycyte or subtypes therein. From an experimental point of view, these markers can be used in conjunction with the morphological criteria listed above to confidently delineate the domain of tanycyte subtypes and/or identify individual tanycytes *in situ*. Also, where available, the relevant Cre or CreERT2 lines could be used to selectively target or lineage-trace the different tanycyte subtypes, so long as the gene is absent from the neighboring parenchymal cells.

## Ageing of tanycytes

Tanycytes proliferate postnatally (see also below) but this capacity seems to wane with increasing age. For example, Lee et al. (2012) and Haan et al. (2013) reported that β-tanycytes can incorporate S-phase marker BrdU, or its analog EDU. However, for β2, this falls dramatically between P7 and P45, whilst even under cumulative labeling paradigms, no BrdU incorporation is seen by 12 months of age in either β1 or β2 tanycytes (Haan et al., 2013). Age-related studies in rats spanning 3–24 months of age, also report that tanycyte numbers decline by almost 30% with increasing age, and whilst immunoreactivity for DARPP32 declines with age, a corresponding increase in GFAP expression is observed (Zoli et al., 1995). Interestingly, it has also been suggested that aged tanycytes become phagocytic and engulf debris produced by nearby degenerating neurons, axons and their myelin sheaths (Brawer and Walsh, 1982). However, the impact of these changes on hypothalamic functioning in general, and any cell-autonomous functions within tanycytes themselves is yet to be determined.

## Metabolic and neuroendocrine functions of tanycytes

Although the specific roles of tanycytes in the hypothalamus are still under much debate, their strategic proximity to, and relationship with fenestrated capillaries, blood brain barrier, axonal nerve terminals, and hypothalamic nuclei that regulate appetite/energy expenditure has placed them in a privileged position to integrate multiple inputs and regulate homeostasis. Some of their prominent roles are discussed below.

Early studies focused on barrier functions of tanycytes, prompted by the finding that when Horse Radish Peroxidase (HRP) is injected into the CSF, for a short period it can diffuse freely into the hypothalamic parenchyma, but not into the Median Eminence (ME). Eventually, HRP reaches ME through the subarachnoid CSF. Conversely, intravenously-injected HRP is locked out of the hypothalamic parenchyma by its blood brain barrier (BBB), but can rapidly diffuse into the ME, and be contained therein (Rodríguez et al., 2005). This and a set of other studies have led to the suggestion that β-tanycytes, particularly β2, communicate relatively freely with plasma through fenestrated capillaries, but maintain a tight barrier between CSF and ME at their apical (cell body/3V ventricular) surface, as well as between ME and the nearby arcuate nucleus. By contrast, α-tanycytes lack this barrier function at the ependymal surface. By inference, it has been suggested that β-tanycytes' barrier functions have created a “three-way exchange interface” within the ME, whereby contents of subarachnoid CSF, neuronal secretions and blood capillaries can mix and communicate. However, the significance of this tripartite communication remains unclear.

β-tanycytes also seem to modulate the release of GnRH into the portal blood thereby regulating LH and FSH release from the pituitary gland (Ojeda et al., 2008). GnRH nerve terminals, whose cell bodies reside in the rostral hypothalamus, converge onto the processes of β-tanycytes, which in turn separate these from the portal capillaries. Upon receipt of the appropriate signals, thought to include Tgfβ, Oestrogen, or CSF-derived IGF-1 (Hiney et al., 1996; Prevot et al., 2000, 2003), tanycytes facilitate GnRH release into the portal blood, possibly by temporarily retracting their processes.

The hypothalamus plays a critical role in the regulation of energy uptake and expenditure, largely through five neuronal nuclei contained within the flanking parenchyma of the 3V—i.e., The dorsomedial (DMN), ventromedial (VMN), Lateral (LHN), the arcuate (Arc) nucleus, and the Paraventricular (PVN). At the core of this regulation lies the Arcuate's anorexigenic and orexigenic neurons, marked by their respective neurotransmitters Pro-opiomelanocortin (POMC), and Neuropeptide Y (NPY)/ and Agouti-related Peptide (AgRP), in addition to other minor neurotransmitters. These neurons sense and integrate a diverse array of anorexigenic and orexigenic metabolic signals such as ghrelin, glucose, and Leptin to promote or suppress appetite (Yeo and Heisler, 2012). Akin to the GnRH gated-release scenario, Langlet et al. (2013) showed that during fasting the barrier function of tanycytes is altered through a VEGF-A dependent mechanism to allow greater vascular permeability and greater contact between circulating metabolites and Arc neurons (Langlet et al., 2013). More recent studies have demonstrated that Leptin, an anorexigenic hormone produced by adipocytes, reaches hypothalamic neurons via tanycytes (Balland et al., 2014). In this context, tanycytes uptake Leptin from portal vessels and release it into the 3V CSF in an ERK-signaling and Leptin-receptor dependent manner, after which Leptin reaches Arc neurons through the walls of the 3V. Interestingly, prolonged exposure of rodents to high-fat diet attenuates this transport mechanism, thereby inducing a “Leptin-deficient” state in hypothalamic neurons (Balland et al., 2014).

Thyroid hormones (TH) are critical regulators of tissue growth and differentiation as well as metabolic homeostasis. Tanycytes also play a critical role in the feedback mechanisms that regulate TH (T4) release, involving the Hypothalamo-Pituitary-Thyroid axis (Fekete and Lechan, 2014). Their main role is to fine-tune the release and bioavailability of Thyrotropin Releasing Hormone (TRH)—derived from TRH-producing neurons of the Paraventricular nucleus (PVN). When released into the portal blood, TRH acts on the pituitary thyrotrophs to stimulate Thyroid Stimulating Hormone β-sub unit (TSHβ) production, which in turn stimulates TH release by the thyroid gland. In turn, circulating TH levels control the levels of TRH production and release via both transcriptional and post-transcriptional mechanisms within PVN's TRH-producing neurons.

Tanycytes (in particular β2) participate in this regulatory pathway via multiple but complementary mechanisms. First, they can actively uptake T4 from the CSF via two transporter molecules, MCT8 and OATP1, and convert T4 into either a more potent form, T3, or an inactive form by expressing the relevant converting enzymes—Deiodinase 2 (DIO2) and DIO3, respectively. Second, within the median eminence, the axonal terminals of TRH-producing neurons release TRH into the extracellular space, but TRH access to portal blood is restricted by the processes of tanycytes, akin to the GnRH gating scenario (above). Thus, it is thought that circulating TH induces cytoskeletal remodeling and retraction of tanycytic processes to allow TRH access to portal blood. Third, tanycytes also produce important enzymes that regulate TRH degradation, chiefly Pyroglutamyl peptidase (PPII; Table 1; Sanchez et al., 2009), thereby regulating the bioavailability of TRH. Fourth, tanycyte-derived T3 is thought to be a major T3 source for TRH-producing neurons themselves, taken up and retrogradely transported via their nerve terminals within ME, as well as for hypothalamic parenchymal neurons via T3 released into the CSF.

This elegant machinery is utilized for multiple adaptive functions, including seasonal (photo-period dependent) and thus long term control of appetite/energy expenditure (Ebling, 2015). Long photoperiods stimulate melatonin and consequently TSHβ release. Binding of TSHβ to its receptor on tanycytes promotes DIO2 expression thus increasing the bioavailability of T3 during long photoperiods (Yasuo et al., 2007), and consequently a lowered energy expenditure status. This is complemented by the production of more T4 and enhancement of retinoic acid signaling pathway during long photoperiods (Shearer et al., 2010), which together act to promote appetite and energy uptake. The net effect is the build up of energy stores (adipose tissue) in preparation for short photoperiods when food may be scarce. Interestingly, in rats, the proliferation of tanycytes increases during short photoperiods and wanes during long photoperiods (Shearer et al., 2012).

Tanycytes, in particular the α subtype, have also been implicated in direct nutrient sensing mechanisms since they express glucose transporter 2 (GLUT2) and occupy a strategic position at the interface of the CSF and the parenchyma where appetite is regulated (García et al., 2003). α-tanycytes also seem to sense plasma glucose levels (Frayling et al., 2011). However, both α- and β1 tanycytes have now been shown to respond to exogenously administered glucose, which stimulates the influx of Calcium (Ca^2+^) ions, ATP release, activation of P2Y1 purinergic receptors and an initiation of internal Ca^2+^ release within tanycytes. This effect then induces a propagative wave of Ca^2+^ signaling across neighboring tanycytes (Dale, 2011). Mechanistically, parallels have been drawn between the way pancreatic β-cells and tanycytes sense glucose, which in the former, involves conversion of glucose to glucose-6-phosphate by glucokinase. Alternatively, glucose could be taken up into tanycytes via GLUT2 transporters or G-protein coupled receptors (GPRCs), expressed by these cells (Bolborea and Dale, 2013). It will be interesting to test whether and how tanycytes respond to other metabolites, such as distinct classes of fatty acids and amino acids, and what is the short and long term significance of their nutrient sensing.

From the foregoing, it is clear that tanycytes and subtypes therein play diverse, yet complimentary and adaptive homeostatic and neuroendocrine roles, involving sensing, shuttling and release of nutrients, hormones, and neurotransmitters.

## Neuro-gliogenic properties of tanycytes

The resemblance of tanycytes to radial glia at gross and ultra-structural levels led researchers, as early as the 1960s, to speculate that hypothalamic tanycytes may act as some sort of progenitor cells in the postnatal brain (Millhouse, 1971). This hypothesis was supported by examining dynamic changes in early characterized neuro-glial progenitor markers such as S100β and Neuron-Specific Enolase (De Vitry et al., 1980). In later years, and with the advent of neurosphere assays (Reynolds and Weiss, 1992), workers were able to derive either neurospheres or establish monolayer cultures of dividing progenitors cells from rodent hypothalamus (Prevot et al., 2003; Markakis et al., 2004; Xu et al., 2005). For example, Markakis et al. (2004) described the production of several hypothalamic neuronal subtypes in these cultures. Some workers even explored the ability of tanycytes to promote and facilitate axonal regeneration, either *in situ* or when transplanted into lesioned spinal cord (Chauvet et al., 1995; Prieto et al., 2000).

A key question for these and subsequent studies was: where are hypothalamic stem/progenitors located? Do these reside within the hypothalamic parenchyma; its ependymal layer; or both? Since hypothalamic tanycytes, ependymal cells, and parenchymal cells reside in tight proximity and their neat separation through microdissection is very challenging, our current assessment of this question is guided by *in vivo* studies.

### Evidence for a parenchymal progenitor cell population

As mentioned above, hypothalamic tanycytes are flanked by neurons/neuronal nuclei that collectively regulate energy uptake and expenditure (Figure 1), the bulk of which are generated prenatally—in mice between E10.5 and E14.5—from hypothalamic ventricular zone cells (Shimada and Nakamura, 1973).

Pierce and Xu (2010) showed that although an acute and total ablation of AgRP neurons causes severe anorexia, their gradual ablation has minimal impact on appetite/energy expenditure, largely because loss of these cells is compensated by *de novo* production of neurons within the hypothalamic parenchyma. Along similar lines, McNay et al. (2012) labeled BrdU-retaining post-mitotic neurons of the Arc during embryogenesis and showed that over half of these are normally lost and then replaced during the first three postnatal months. Using injected viral vectors to lineage trace Sox2-expresing cells that are normally scattered within the adult hypothalamic parenchyma, Li et al. (2012) demonstrated that over long survival periods, virally transduced cells can generate both neurons and glial cells *in vivo*. They concluded that Sox2+ cells constitute a neural stem cell population. However, Sox2 is also strongly expressed by the nearby 3V ependymal cells, including tanycytes (Li et al., 2012; Haan et al., 2013).

In more recent studies, Robins et al. (2013b) showed that a significant number of parenchymal Sox2-expressing cells co-express the neuroglia marker, NG2 (Dimou and Gallo, 2015), and a limited number of neurons can be derived from NG2-lineage traced cells (Robins et al., 2013b). Furthermore, NG2-expressing cells residing at the periphery of hypothalamic parenchyma become activated and replace more medial NG2+ cells following their focal depletion through intracerebroventricular infusion of a mitotic inhibitor, Cytosine arabinoside (AraC) (Robins et al., 2013c).

Combined, these studies demonstrate that the postnatal hypothalamic parenchyma contains one or more set of bi- or multi-potent progenitors cells. However, their high mitotic activity and their punctate dispersal throughout the parenchyma argues against a “stem cell” status, since, stem cells usually have low rate of division and are restricted to defined micro-niches.

### Neurogliogenic potential of tanycytes

The ability of 3V ependymo-tanycytes to incorporate tritiated-thymidine or BrdU was amongst the earliest indicators that tanycytes may have neurogenic properties. The exact rate of cell division in tanycytes has not been determined, but clearly, short pulses of BrdU used to label neural stem/progenitors in the hippocampal subgranular zone (SGZ) or the lateral subventricular zone (SVZ) is insufficient to label tanycytes. Hence, early studies resorted to either multiple intraperitoneal injections or by direct intracerebroventricular infusion of BrdU, with the latter resulting in a greater number of labeled cells (Kokoeva et al., 2005, 2007; Pérez-Martín et al., 2010). More recently, BrdU, or its analog EDU, have also been successfully applied cumulatively via drinking water over a number of days (Haan et al., 2013) to label tanycytes.

Icv-based pulse chase studies showed that with time, BrdU-labeled cells emerge within the hypothalamic parenchyma, where a subset differentiated into functionally integrated neurons, and astroglial cells (Kokoeva et al., 2005). Remarkably, BrdU incorporation could be enhanced by intraventricular application of several growth factors, including BDNF, CNTF, FGF2 (formerly basic FGF), and IGF-I (Pencea et al., 2001; Xu et al., 2005; Kokoeva et al., 2007; Pérez-Martín et al., 2010).

Taken together, this was strong evidence that an ependymo-tanycytic population gives rise to new neurons and glial cells in the postnatal brain. However, the exact identity and location of this neurogenic cell population within the ependyma remained unresolved, but more recent genetic lineage tracing studies have shed more light on this question.

### Direct lineage tracing of tanycytes

Lineage tracing studies have proved instrumental in delineating the fate and potential of diverse neural stem/progenitor cells in the postnatal and adult CNS (e.g., Ganat et al., 2006; Rivers et al., 2008; Beckervordersandforth et al., 2010). Typically, these rely on the constitutive activation of a marker protein (fluorescent or otherwise) at desired time points in the stem/progenitor cell population in question, and the subsequent monitoring of the division, migration and differentiation pattern of their progeny. This is achieved by using double transgenic mice harboring a CreERT2 transgene driven by a gene specific promoter, as well as a cassette encoding marker proteins (LacZ, YFP or tomato-dsred etc.) placed within the Rosa-26R allele but kept silent by upstream LoxP-STOP-LoxP sequences (available through jax labs; www.jax.org). Application of tamoxifen to these mice causes a nuclear translocation of CreERT2 protein to delete the STOP codon and activate the reporter marker, only in cells expressing the gene of interest.

Lee et al. (2012) used nestin-creERT2 mice to lineage trace β2-tanycytes at postnatal day 4 (P4) and analyzed these at P7 and P35 (Lee et al., 2012). Lineage-traced cells were subsequently found mostly in the median eminence, with minor contributions to parenchymal nuclei (DMN, VMN, Arc, and LHN). Lineage traced ME cells differentiated mostly into neurons, of multiple subtypes—GABAergic, POMC- and NPY-expressing cells, but not glial cells. Moreover, the neurons were retained for up to a month, demonstrating that they are not a transient population. Subsequent functional analysis showed that suppression of ME neurogenesis is protective against high-fat diet induced obesity, and therefore postnatally generated ME neurons play a crucial role in energy expenditure. Interestingly, although nestin continues to be expressed by tanycytes into adulthood and neurospheres can be derived from this population *in vitro* (Bennett et al., 2009), there are no reports of successful nestin-creERT2 lineage tracing of tancytes in the adult hypothalamus *in vivo*.

Haan et al. (2013) took advantage of earlier reports that Fgf10 is expressed specifically by β(1- and 2-) tanycytes (Hajihosseini et al., 2008) but not the neighboring parenchymal cells, and lineage traced these *in vivo*—indirectly using Fgf10-lacZ (Kelly et al., 2001), and directly using Fgf10-creERT2 mice (El Agha et al., 2012). They showed that although Fgf10-expressing (Fgf10+) tanycytes' major contribution is to the Arc nucleus, lineage traced cells also populate the VMN, DMN, and LHN. The Arc progeny includes orexigenic (NPY+) neurons (Haan et al., 2013) and it remains to be determined whether or not anorexigenic neurons are also generated by Fgf10+ tanycytes. However, a rare contribution to parenchymal astrocytes was also observed. Direct lineage tracing at P28 and P57, analyzed between 4 and 40 days later further revealed that Fgf10-expressing β-tanycytes themselves amplify in number within the ependymal zone and their parenchymal progeny continue to proliferate within the parenchyma itself. Remarkably, lineage traced cells appeared to disperse rostrally as well as coronally within the hypothalamic parenchyma (Haan et al., 2013).

Robins et al. (2013a) lineage traced GLAST-expressing cells, normally found in the α-tanycyte domain as well as some parenchymal astrocytes (Robins et al., 2013a). They reported an impressive expansion and retention of lineage-traced α-tanycytes with some cells entering the domain of β-tanycytes during long time-point analysis, suggestive of a remarkable fluidity within the ependymal cell layer. However, the major parenchymal contribution of P42–P60 lineage-traced cells was to the astroglial lineage, accompanied by only a few neurons. In view of the inherent GLAST expression by some parenchymal cells and existence of inherent parenchymal progenitor cells (discussed above), the absolute parenchymal contribution of GLAST-expressing α-tanycytes remains to be determined. Interestingly, GLAST-expressing tanycytes were also found to respond to exogenous FGF2 stimulation, which is likely mediated by an FGF-receptor other than FGFR1 and FGFR2, since their expression in the hypothalamic ependyma is restricted to β-tanycytes (Allen brain atlas; Table 1). A micro-dissection approach was also used to demonstrate that α-tanycytes but not β-tanycytes have neurospherogenic capacity *in vitro* (Robins et al., 2013a).

In more recent studies, Rax-CreERT2 mice have been used to lineage trace P7 to P50 α- and β-tanycytes, since depending on bregma co-ordinates, Rax is expressed by both tanycyte subtypes. Results show clear emergence of parenchymal lineage traced cells that largely resemble glial cells or poorly-differentiated neural progenitors (Pak et al., 2014).

Combined, the above studies have now conclusively shown that postnatal tanycytes have a neuro-gliogenic capacity, with β-tanycytes being more neurogenic, whilst α-tanycytes generating mostly glial cells. Furthermore, their strong resemblance to radial glial cells, their discrete arrangement within the 3V ependma and their expression of neural stem/progenitor cell markers gives credence to the idea that tanycytes act as bona fide stem cells in the postnatal hypothalamus. If so, then tanycytes could be the ultimate source of parenchymal progenitor cells described above and sit at the top of the hierarchy of hypothalamic neurogenesis. Thus, the definition of “hypothalamic neurogenesis” is pivotal when measuring external influences such as treatment with high-fat diet i.e., It is important to determine whether such metabolites act on tanycytes, their progeny, or both.

## Concluding remarks and future perspectives

In comparison to other cell types in the CNS (likes of pericytes, NG2-glia and microglia), our knowledge of tanycytes is still rudimentary. In particular, we know very little about their anterio-posterior regionalization. Given their diverse functions (highlighted above), a set of pertaining questions ensues:

- If tanycytes constitute a neural stem cell population, what are the endogenous factors and genetic regulators driving their development (survival, proliferation, and differentiation), or maintenance with their niche? Are these comparable to those that regulate postnatal and adult neural stem cells in SGZ and SVZ?
- What are the intermediate steps of neurogenesis downstream of tanycytes? Is an intermediate or transient amplifying cell population involved, and how are particular neuronal subtypes produced? Do these properties change with age?
- How heterogeneous are tanycytes? For example, are specific populations set aside for barrier and transport functions vs. neurogenesis, or does a single tanycyte cell population multi-task? If there is heterogeneity, what factors establish this functional diversification? If not, are the chemosensory and homeostatic functions directly linked to neurogenesis?
- Tanycytes are found in the human hypothalamus (Flament-Durand and Brion, 1985) but are these more or less numerous than in rodents, and are the functions assigned to rodent tanycytes conserved in human? This is an important consideration, if the tanycytes and their derivatives (parenchymal neurons and progenitor cells) are to be exploited to tackle neural malfunctioning in the hypothalamus that seems to underlie eating disorders.

Fortunately, with the plethora of novel experimental tools and protocols at our disposal—ranging from novel transgenic animal models, gene transducing viruses, and cross-synaptic viral tracers to optogenetics—we are in an exciting era to answer these and other pertinent questions thereby learning more about the biology of tanycytes.

### Conflict of interest statement

The authors declare that the research was conducted in the absence of any commercial or financial relationships that could be construed as a potential conflict of interest.

## References

[B1] Altman J. Bayer S. A. (1978). Development of the diencephalon in the rat. III. Ontogeny of the specialized ventricular linings of the hypothalamic third ventricle. J. Comp. Neurol. 182(4 Pt 2), 995–1015. 10.1002/cne.901820513 730854

[B2] Balland E. Dam J. Langlet F. Caron E. Steculorum S. Messina A.. (2014). Hypothalamic tanycytes are an ERK-gated conduit for leptin into the brain. Cell Metab. 19, 293–301. 10.1016/j.cmet.2013.12.015 24506870 PMC3936883

[B3] Batailler M. Mullier A. Sidibe A. Delagrange P. Prévot V. Jockers R.. (2012). Neuroanatomical distribution of the orphan GPR50 receptor in adult sheep and rodent brains. J. Neuroendocrinol. 24, 798–808. 10.1111/j.1365-2826.2012.02274.x 22512326

[B4] Beckervordersandforth R. Tripathi P. Ninkovic J. Bayam E. Lepier A. Stempfhuber B.. (2010). *In vivo* fate mapping and expression analysis reveals molecular hallmarks of prospectively isolated adult neural stem cells. Cell stem cell 7, 744–758. 10.1016/j.stem.2010.11.017 21112568

[B5] Belluardo N. Wu G. Mudo G. Hansson A. C. Pettersson R. Fuxe K. (1997). Comparative localization of fibroblast growth factor receptor-1, -2, and -3 mRNAs in the rat brain: *in situ* hybridization analysis. J. Comp. Neurol. 379, 226–246. 9050787

[B6] Bennett L. Yang M. Enikolopov G. Iacovitti L. (2009). Circumventricular organs: a novel site of neural stem cells in the adult brain. Mol. Cell. Neurosci. 41, 337–347. 10.1016/j.mcn.2009.04.007 19409493 PMC2697272

[B7] Berger U. V. Hediger M. A. (2001). Differential distribution of the glutamate transporters GLT-1 and GLAST in tanycytes of the third ventricle. J. Comp. Neurol. 433, 101–114. 10.1002/cne.1128 11283952

[B8] Bolborea M. Dale N. (2013). Hypothalamic tanycytes: potential roles in the control of feeding and energy balance. Trends Neurosci. 36, 91–100. 10.1016/j.tins.2012.12.008 23332797 PMC3605593

[B9] Brawer J. R. Walsh R. J. (1982). Response of tanycytes to aging in the median eminence of the rat. Am. J. Anat. 163, 247–256. 10.1002/aja.1001630305 7091013

[B10] Chauvet N. Parmentier M. L. Alonso G. (1995). Transected axons of adult hypothalamo-neurohypophysial neurons regenerate along tanycytic processes. J. Neurosci. Res. 41, 129–144. 10.1002/jnr.490410115 7674374

[B11] Cheng M. F. (2013). Hypothalamic neurogenesis in the adult brain. Front. Neuroendocrinol. 34, 167–178. 10.1016/j.yfrne.2013.05.001 23684668

[B12] Coll A. P. Yeo G. S. (2013). The hypothalamus and metabolism: integrating signals to control energy and glucose homeostasis. Curr. Opin. Pharmacol. 13, 970–976. 10.1016/j.coph.2013.09.010 24075719

[B13] Dale N. (2011). Purinergic signaling in hypothalamic tanycytes: potential roles in chemosensing. Semin. Cell Dev. Biol. 22, 237–244. 10.1016/j.semcdb.2011.02.024 21396904

[B14] De Juan Romero C. Borrell V. (2015). Coevolution of radial glial cells and the cerebral cortex. Glia 63, 1303–1319. 10.1002/glia.22827 25808466 PMC5008138

[B15] De Vitry F. Picart R. Jacque C. Legault L. Dupouey P. Tixier-Vidal A. (1980). Presumptive common precursor for neuronal and glial cell lineages in mouse hypothalamus. Proc. Natl. Acad. Sci. U.S.A. 77, 4165–4169. 10.1073/pnas.77.7.4165 7001456 PMC349791

[B16] Dimou L. Gallo V. (2015). NG2-glia and their functions in the central nervous system. Glia 63, 1429–1451. 10.1002/glia.22859 26010717 PMC4470768

[B17] Ebling F. J. (2015). Hypothalamic control of seasonal changes in food intake and body weight. Front. Neuroendocrinol. 37, 97–107. 10.1016/j.yfrne.2014.10.003 25449796

[B18] El Agha E. Al Alam D. Carraro G. Mackenzie B. Goth K. De Langhe S. P.. (2012). Characterization of a Novel Fibroblast Growth Factor 10 (Fgf10) Knock-In Mouse Line to Target Mesenchymal Progenitors during Embryonic Development. PLoS ONE 7:e38452. 10.1371/journal.pone.0038452 22719891 PMC3374781

[B19] Fekete C. Lechan R. M. (2014). Central regulation of hypothalamic-pituitary-thyroid axis under physiological and pathophysiological conditions. Endocr. Rev. 35, 159–194. 10.1210/er.2013-1087 24423980 PMC3963261

[B20] Fekete C. Mihály E. Herscovici S. Salas J. Tu H. Larsen P. R.. (2000). DARPP-32 and CREB are present in type 2 iodothyronine deiodinase-producing tanycytes: implications for the regulation of type 2 deiodinase activity. Brain Res. 862, 154–161. 10.1016/S0006-8993(00)02105-3 10799680

[B21] Flament-Durand J. Brion J. P. (1985). Tanycytes: morphology and functions: a review. Int. Rev. Cytol. 96, 121–155. 10.1016/S0074-7696(08)60596-3 2416706

[B22] Frayling C. Britton R. Dale N. (2011). ATP-mediated glucosensing by hypothalamic tanycytes. J. Physiol. 589(Pt 9), 2275–2286. 10.1113/jphysiol.2010.202051 21486800 PMC3098703

[B23] Ganat Y. M. Silbereis J. Cave C. Ngu H. Anderson G. M. Ohkubo Y.. (2006). Early postnatal astroglial cells produce multilineage precursors and neural stem cells *in vivo*. J. Neurosci. 26, 8609–8621. 10.1523/JNEUROSCI.2532-06.2006 16914687 PMC6674357

[B24] García M. Millán C. Balmaceda-Aguilera C. Castro T. Pastor P. Montecinos H.. (2003). Hypothalamic ependymal-glial cells express the glucose transporter GLUT2, a protein involved in glucose sensing. J. Neurochem. 86, 709–724. 10.1046/j.1471-4159.2003.01892.x 12859684

[B25] Guadaño-Ferraz A. Obregón M. J. St. Germain D. L. Bernal J. (1997). The type 2 iodothyronine deiodinase is expressed primarily in glial cells in the neonatal rat brain. Proc. Natl. Acad. Sci. U.S.A. 94, 10391–10396. 9294221 10.1073/pnas.94.19.10391 PMC23373

[B26] Haan N. Goodman T. Najdi-Samiei A. Stratford C. M. Rice R. El Agha E.. (2013). Fgf10-expressing tanycytes add new neurons to the appetite/energy-balance regulating centers of the postnatal and adult hypothalamus. J. Neurosci. 33, 6170–6180. 10.1523/JNEUROSCI.2437-12.2013 23554498 PMC3736310

[B27] Hajihosseini M. K. De Langhe S. Lana-Elola E. Morrison H. Sparshott N. Kelly R.. (2008). Localization and fate of Fgf10-expressing cells in the adult mouse brain implicate Fgf10 in control of neurogenesis. Mol. Cell. Neurosci. 37, 857–868. 10.1016/j.mcn.2008.01.008 18329286

[B28] Han Y. G. Spassky N. Romaguera-Ros M. Garcia-Verdugo J. M. Aguilar A. Schneider-Maunoury S.. (2008). Hedgehog signaling and primary cilia are required for the formation of adult neural stem cells. Nat. Neurosci. 11, 277–284. 10.1038/nn2059 18297065

[B29] Helfer G. Ross A. W. Russell L. Thomson L. M. Shearer K. D. Goodman T. H.. (2012). Photoperiod regulates vitamin A and Wnt/beta-catenin signaling in F344 rats. Endocrinology 153, 815–824. 10.1210/en.2011-1792 22210746

[B30] Hiney J. K. Srivastava V. Nyberg C. L. Ojeda S. R. Dees W. L. (1996). Insulin-like growth factor I of peripheral origin acts centrally to accelerate the initiation of female puberty. Endocrinology 137, 3717–3728. 8756538 10.1210/endo.137.9.8756538

[B31] Kelly R. G. Brown N. A. Buckingham M. E. (2001). The arterial pole of the mouse heart forms from Fgf10-expressing cells in pharyngeal mesoderm. Dev. Cell 1, 435–440. 10.1016/S1534-5807(01)00040-5 11702954

[B32] Kokoeva M. V. Yin H. Flier J. S. (2005). Neurogenesis in the hypothalamus of adult mice: potential role in energy balance. Science 310, 679–683. 10.1126/science.1115360 16254185

[B33] Kokoeva M. V. Yin H. Flier J. S. (2007). Evidence for constitutive neural cell proliferation in the adult murine hypothalamus. J. Comp. Neurol. 505, 209–220. 10.1002/cne.21492 17853440

[B34] Langlet F. (2014). Tanycytes: a gateway to the metabolic hypothalamus. J. Neuroendocrinol. 26, 753–760. 10.1111/jne.12191 25131689

[B35] Langlet F. Mullier A. Bouret S. G. Prevot V. Dehouck B. (2013). Tanycyte-like cells form a blood-cerebrospinal fluid barrier in the circumventricular organs of the mouse brain. J. Comp. Neurol. 521, 3389–3405. 10.1002/cne.23355 23649873 PMC3973970

[B36] Lee D. A. Bedont J. L. Pak T. Wang H. Song J. Miranda-Angulo A.. (2012). Tanycytes of the hypothalamic median eminence form a diet-responsive neurogenic niche. Nat. Neurosci. 15, 700–702. 10.1038/nn.3079 22446882 PMC3380241

[B37] Lee D. A. Blackshaw S. (2014). Feed your head: neurodevelopmental control of feeding and metabolism. Annu. Rev. Physiol. 76, 197–223. 10.1146/annurev-physiol-021113-170347 24274739 PMC4512170

[B38] Li J. Tang Y. Cai D. (2012). IKKbeta/NF-kappaB disrupts adult hypothalamic neural stem cells to mediate a neurodegenerative mechanism of dietary obesity and pre-diabetes. Nat. Cell Biol. 14, 999–1012. 10.1038/ncb2562 22940906 PMC3463771

[B39] Ma M. S. Brouwer N. Wesseling E. Raj D. van der Want J. Boddeke E.. (2015). Multipotent stem cell factor UGS148 is a marker for tanycytes in the adult hypothalamus. Mol. Cell. Neurosci. 65, 21–30. 10.1016/j.mcn.2015.02.002 25662290

[B40] Markakis E. A. Palmer T. D. Randolph-Moore L. Rakic P. Gage F. H. (2004). Novel neuronal phenotypes from neural progenitor cells. J. Neurosci. 24, 2886–2897. 10.1523/JNEUROSCI.4161-03.2004 15044527 PMC3242437

[B41] McNay D. E. Briancon N. Kokoeva M. V. Maratos-Flier E. Flier J. S. (2012). Remodeling of the arcuate nucleus energy-balance circuit is inhibited in obese mice. J. Clin. Invest. 122, 142–152. 10.1172/JCI43134 22201680 PMC3248278

[B42] Millhouse O. E. (1971). A Golgi study of third ventricle tanycytes in the adult rodent brain. Z. Zellforsch. Mikrosk. Anat. 121, 1–13. 10.1007/BF00330913 5112429

[B43] Miranda-Angulo A. L. Byerly M. S. Mesa J. Wang H. Blackshaw S. (2014). Rax regulates hypothalamic tanycyte differentiation and barrier function in mice. J. Comp. Neurol. 522, 876–899. 10.1002/cne.23451 23939786 PMC3947139

[B44] Mori T. Tanaka K. Buffo A. Wurst W. Kühn R. Götz M. (2006). Inducible gene deletion in astroglia and radial glia–a valuable tool for functional and lineage analysis. Glia 54, 21–34. 10.1002/glia.20350 16652340

[B45] Morita S. Furube E. Mannari T. Okuda H. Tatsumi K. Wanaka A.. (2015). Heterogeneous vascular permeability and alternative diffusion barrier in sensory circumventricular organs of adult mouse brain. Cell Tissue Res. 10.1007/s00441-015-2207-7. [Epub ahead of print]. 26048259

[B46] Ojeda S. R. Lomniczi A. Sandau U. S. (2008). Glial-gonadotrophin hormone (GnRH) neurone interactions in the median eminence and the control of GnRH secretion. J. Neuroendocrinol. 20, 732–742. 10.1111/j.1365-2826.2008.01712.x 18601696

[B47] Pak T. Yoo S. Miranda-Angulo A. L. Wang H. Blackshaw S. (2014). Rax-CreERT2 knock-in mice: a tool for selective and conditional gene deletion in progenitor cells and radial glia of the retina and hypothalamus. PLoS ONE 9:e90381. 10.1371/journal.pone.0090381 24699247 PMC3974648

[B48] Pencea V. Bingaman K. D. Wiegand S. J. Luskin M. B. (2001). Infusion of brain-derived neurotrophic factor into the lateral ventricle of the adult rat leads to new neurons in the parenchyma of the striatum, septum, thalamus, and hypothalamus. J. Neurosci. 21, 6706–6717. 11517260 10.1523/JNEUROSCI.21-17-06706.2001 PMC6763082

[B49] Pérez-Martín M. Cifuentes M. Grondona J. M. Lopez-Avalos M. D. Gómez-Pinedo U. García-Verdugo J. M.. (2010). IGF-I stimulates neurogenesis in the hypothalamus of adult rats. Eur. J. Neurosci. 31, 1533–1548. 10.1111/j.1460-9568.2010.07220.x 20525067

[B50] Peruzzo B. Pastor F. E. Blázquez J. L. Amat P. Rodríguez E. M. (2004). Polarized endocytosis and transcytosis in the hypothalamic tanycytes of the rat. Cell Tissue Res. 317, 147–164. 10.1007/s00441-004-0899-1 15221441

[B51] Peruzzo B. Pastor F. E. Blázquez J. L. Schöbitz K. Peláez B. Amat P.. (2000). A second look at the barriers of the medial basal hypothalamus. Exp. Brain Res. 132, 10–26. 10.1007/s002219900289 10836632

[B52] Pierce A. A. Xu A. W. (2010). De novo neurogenesis in adult hypothalamus as a compensatory mechanism to regulate energy balance. J. Neurosci. 30, 723–730. 10.1523/JNEUROSCI.2479-09.2010 20071537 PMC3080014

[B53] Prevot V. Bouret S. Croix D. Takumi T. Jennes L. Mitchell V.. (2000). Evidence that members of the TGFbeta superfamily play a role in regulation of the GnRH neuroendocrine axis: expression of a type I serine-threonine kinase receptor for TGRbeta and activin in GnRH neurones and hypothalamic areas of the female rat. J. Neuroendocrinol. 12, 665–670. 10.1046/j.1365-2826.2000.00508.x 10849211

[B54] Prevot V. Cornea A. Mungenast A. Smiley G. Ojeda S. R. (2003). Activation of erbB-1 signaling in tanycytes of the median eminence stimulates transforming growth factor beta1 release via prostaglandin E2 production and induces cell plasticity. J. Neurosci. 23, 10622–10632. 14627647 10.1523/JNEUROSCI.23-33-10622.2003 PMC6740908

[B55] Prieto M. Chauvet N. Alonso G. (2000). Tanycytes transplanted into the adult rat spinal cord support the regeneration of lesioned axons. Exp. Neurol. 161, 27–37. 10.1006/exnr.1999.7223 10683271

[B56] Reynolds B. A. Weiss S. (1992). Generation of neurons and astrocytes from isolated cells of the adult mammalian central nervous system. Science 255, 1707–1710. 10.1126/science.1553558 1553558

[B57] Rivers L. E. Young K. M. Rizzi M. Jamen F. Psachoulia K. Wade A.. (2008). PDGFRA/NG2 glia generate myelinating oligodendrocytes and piriform projection neurons in adult mice. Nat. Neurosci. 11, 1392–1401. 10.1038/nn.2220 18849983 PMC3842596

[B58] Robins S. C. Stewart I. McNay D. E. Taylor V. Giachino C. Goetz M.. (2013a). alpha-Tanycytes of the adult hypothalamic third ventricle include distinct populations of FGF-responsive neural progenitors. Nat. Commun. 4:2049. 10.1038/ncomms3049 23804023

[B59] Robins S. C. Trudel E. Rotondi O. Liu X. Djogo T. Kryzskaya D.. (2013b). Evidence for NG2-glia derived, adult-born functional neurons in the hypothalamus. PLoS ONE 8:e78236. 10.1371/journal.pone.0078236 24205170 PMC3812154

[B60] Robins S. C. Villemain A. Liu X. Djogo T. Kryzskaya D. Storch K. F.. (2013c). Extensive regenerative plasticity among adult NG2-glia populations is exclusively based on self-renewal. Glia 61, 1735–1747. 10.1002/glia.22554 23918524

[B61] Rodríguez E. M. Blázquez J. L. Guerra M. (2010). The design of barriers in the hypothalamus allows the median eminence and the arcuate nucleus to enjoy private milieus: the former opens to the portal blood and the latter to the cerebrospinal fluid. Peptides 31, 757–776. 10.1016/j.peptides.2010.01.003 20093161

[B62] Rodríguez E. M. Blázquez J. L. Pastor F. E. Peláez B. Peña P. Peruzzo B.. (2005). Hypothalamic tanycytes: a key component of brain-endocrine interaction. Int. Rev. Cytol. 247, 89–164. 10.1016/S0074-7696(05)47003-5 16344112

[B63] Salvatierra J. Lee D. A. Zibetti C. Duran-Moreno M. Yoo S. Newman E. A.. (2014). The LIM homeodomain factor Lhx2 is required for hypothalamic tanycyte specification and differentiation. J. Neurosci. 34, 16809–16820. 10.1523/JNEUROSCI.1711-14.2014 25505333 PMC4261103

[B64] Sánchez E. Vargas M. A. Singru P. S. Pascual I. Romero F. Fekete C.. (2009). Tanycyte pyroglutamyl peptidase II contributes to regulation of the hypothalamic-pituitary-thyroid axis through glial-axonal associations in the median eminence. Endocrinology 150, 2283–2291. 10.1210/en.2008-1643 19179432 PMC2671897

[B65] Severi I. Carradori M. R. Lorenzi T. Amici A. Cinti S. Giordano A. (2012). Constitutive expression of ciliary neurotrophic factor in mouse hypothalamus. J. Anat. 220, 622–631. 10.1111/j.1469-7580.2012.01498.x 22458546 PMC3390515

[B66] Shearer K. D. Goodman T. H. Ross A. W. Reilly L. Morgan P. J. McCaffery P. J. (2010). Photoperiodic regulation of retinoic acid signaling in the hypothalamus. J. Neurochem. 112, 246–257. 10.1111/j.1471-4159.2009.06455.x 19860856

[B67] Shearer K. D. Stoney P. N. Nanescu S. E. Helfer G. Barrett P. Ross A. W.. (2012). Photoperiodic expression of two RALDH enzymes and the regulation of cell proliferation by retinoic acid in the rat hypothalamus. J. Neurochem. 122, 789–799. 10.1111/j.1471-4159.2012.07824.x 22681644

[B68] Shimada M. Nakamura T. (1973). Time of neuron origin in mouse hypothalamic nuclei. Exp. Neurol. 41, 163–173. 10.1016/0014-4886(73)90187-8 4743483

[B69] Sousa-Ferreira L. de Almeida L. P. Cavadas C. (2014). Role of hypothalamic neurogenesis in feeding regulation. Trends Endocrinol. Metab. 25, 80–88. 10.1016/j.tem.2013.10.005 24231724

[B70] Tu H. M. Kim S. W. Salvatore D. Bartha T. Legradi G. Larsen P. R.. (1997). Regional distribution of type 2 thyroxine deiodinase messenger ribonucleic acid in rat hypothalamus and pituitary and its regulation by thyroid hormone. Endocrinology 138, 3359–3368. 10.1210/en.138.8.3359 9231788

[B71] Walsh C. Cepko C. L. (1993). Clonal dispersion in proliferative layers of developing cerebral cortex. Nature 362, 632–635. 10.1038/362632a0 8464513

[B72] Wei L. C. Shi M. Chen L. W. Cao R. Zhang P. Chan Y. S. (2002). Nestin-containing cells express glial fibrillary acidic protein in the proliferative regions of central nervous system of postnatal developing and adult mice. Brain Res. Dev. Brain Res. 139, 9–17. 10.1016/S0165-3806(02)00509-6 12414089

[B73] Xu Y. Tamamaki N. Noda T. Kimura K. Itokazu Y. Matsumoto N.. (2005). Neurogenesis in the ependymal layer of the adult rat 3rd ventricle. Exp. Neurol. 192, 251–264. 10.1016/j.expneurol.2004.12.021 15755543

[B74] Yasuo S. Watanabe M. Iigo M. Nakamura T. J. Watanabe T. Takagi T.. (2007). Differential response of type 2 deiodinase gene expression to photoperiod between photoperiodic Fischer 344 and nonphotoperiodic Wistar rats. Am. J. Physiol. Regul. Integr. Comp. Physiol. 292, R1315–R1319. 10.1152/ajpregu.00396.2006 17110533

[B75] Yeo G. S. Heisler L. K. (2012). Unraveling the brain regulation of appetite: lessons from genetics. Nat. Neurosci. 15, 1343–1349. 10.1038/nn.3211 23007189

[B76] Zoli M. Ferraguti F. Frasoldati A. Biagini G. Agnati L. F. (1995). Age-related alterations in tanycytes of the mediobasal hypothalamus of the male rat. Neurobiol. Aging 16, 77–83. 10.1016/0197-4580(95)80010-O 7723939

